# Differences in the insect fauna associated to a monocultural pasture and a silvopasture in Southeastern Brazil

**DOI:** 10.1038/s41598-020-68973-5

**Published:** 2020-07-21

**Authors:** Iris Guedes Paiva, Alexander Machado Auad, Bruno Antonio Veríssimo, Luís Cláudio Paterno Silveira

**Affiliations:** 1 Programa de Pós-Graduação em Entomologia, Universidade Federal de Lavras-UFLA, Campus Universitário, Caixa Postal 3037, Lavras, MG CEP 37200-900 Brazil; 2 Embrapa Gado de Leite/CNPGL, Juiz de Fora, MG CEP 36.038-330 Brazil; 3 Programa de Pós-Graduação em Comportamento e Biologia Animal, Universidade Federal de Juiz de Fora, Juiz de Fora, MG Brasil; 4 Departmento de Entomologia, Universidade Federal de Lavras-UFLA, Campus Universitário, Caixa Postal 3037, Lavras, MG CEP 37200-900 Brazil

**Keywords:** Ecology, Biodiversity

## Abstract

A major challenge for global agriculture is the reduction of the environmental impacts caused by meat and dairy production, and the conversion of monocultural pastures to silvopastoral systems has emerged as an important ally in this process. In order to understand the effects of this conversion we analysed 4 years of sampling of the insect fauna from a conventional monocultural pasture and a silvopastoral system in Minas Gerais, Brazil. We aimed to determine whether the changes caused by the conversion affected the abundance, richness and diversity of the insect orders found in the two systems. Total abundance, richness and diversity did not differ between the two systems, but we detected a significant difference in community composition. Several insect orders showed differences in either abundance, richness or diversity between the two systems, and several families of Hymenoptera, which contains pollinators and natural enemies, showed important increases in the silvopasture. Conversion of monocultural pastures to silvopastures can have important consequences on insect fauna involved in essential ecosystem functions, and the implementation of silvopastures at larger scales has the potential to benefit biodiversity conservation and ecosystem service provision at the landscape scale.

## Introduction

Bovine livestock is one of the most important sectors of Brazilian agribusiness, and consequently of Brazil’s national economy^1^. Over 80% of the herd depends on pastures as its major source of food. Pastures are a practical and economically efficient form of producing and offering food to cattle, since they are resistant to fluctuations in grain prices, guaranteeing relatively low costs of production^2,3^. An important characteristic of the dynamics of Brazilian pastures has been the substitution of native pastures, composed of native grassland ecosystems in the Cerrado and Pampas, with pastures sown with exotic grass species, especially from the genus *Brachiaria*, which currently constitute over 50% Brazilian pastures^2^.

Intensive management of agricultural ecosystems and inappropriate pasture management, such as a lack of nutrient addition, result in the simplification of biological communities, and are the main cause of pasture degradation^4,5^. In the search for production systems that minimise the effects of land degradation and increase the profit obtained by farmers, agroforests have emerged as an important alternative^6^. Agroforests are increasingly used in Latin America to enhance biodiversity and the provision of ecosystem services. The adoption of agroforests in pastures, in the form of silvopastoral systems, creates an environment that buffers temperature extremes and is more comfortable for the animals, reduces erosion from runoff and wind and increases soil biomass, thus contributing to nutrient cycling^7^. Furthermore, combining pastures with trees creates a diversity of microclimates that increases insect diversity^8^.

The effect of conversion to silvopastoral systems on specific insect groups has been studied previously^9–14^, and it is known that conversion of a monoculture of *Brachiaria decumbens* to a silvopasture increases Hymenoptera diversity^15^. Inventories of insect diversity in ecosystems allow us to assess how to prevent or remedy the impacts of environmental change. Therefore, with a change in plant diversity and composition, as well as ecosystem stability, insect abundance and diversity change, and serve as indicators of change^16^. In Brazil, studies that assess insect diversity in pastures are sparse, and there are no inventories that describe the complete insect fauna in silvopastures and *B. decumbens* monocultures that could allow us to generalise conclusions on the effects of conversion.

A common method to detect and monitor changes in biodiversity caused by human activities is to use species or groups that act as bioindicators of environmental degradation^17^. Insects, because of their high diversity and sensitivity to disturbance, have been commonly used as bioindicators. The order Hymenoptera is among the most diverse insect groups, with over 154,000 described species^18^. Furthermore, species of Hymenoptera are potential bioindicators, since the abundance and richness of many of the families are spatially and temporally variable and these variations are correlated to changes in environmental structure and diversity of other organisms^11,15,19–22^. Another important order in pastures is Hemiptera. The implementation of exotic pasture monocultures in Brazil has resulted, over time, in an important increase in the abundance of several species in this order^23,24^, especially those with a phytophagous habit. A consequence of this increase has been a greater incidence of pests in Brazilian pastures that cause important losses in the production of both dairy and beef livestock^25^, and are also an important cause of the acceleration of pasture degradation^26^.

With this in mind, the aim of this study was: (i) to describe and compare the insect fauna in silvopastoral systems and *B. decumbens* monocultures; (ii) to assess whether the environmental changes caused by the conversion of *B. decumbens* monocultures to silvopastures alter the abundance, richness and diversity of insects in the orders Hymenoptera and Hemiptera, as well as in the major functional groups, with a view of identifying biological indicators.

## Results

### Overall insect fauna

We collected a total of 94,613 individuals from 14 orders. Hemiptera was the most abundant order, followed by Hymenoptera, Diptera and Coleoptera, which together made up 97% of the individuals sampled (Table 1). Total abundance in the silvopastural system was 48,338 from 1,087 morphospecies, and 520 morphospecies were exclusive to this system. In the pasture monoculture we collected a total of 46,275 individuals from 932 morphospecies, and 363 morphospecies were exclusive to this system. No significant differences between the monocultural and silvopastoral systems were found in abundance or species richness (Table 1). Shannon’s H′ was higher in the monoculture (Table 1).

**Table 1:** Abundance, richness and Shannon’s H′ diversity of the insects collected in the silvopasture (S) and *Brachiaria decumbens* monoculture (M).

Order	Abundance				Richness				Shannon’s H′	
	S	M	V	*p* value	S	M	V	*p* value	S	M
Hemiptera	23,133	18,571	1,286.5	0.1522	111	89	1,033.5	0.0565	1.143	1.219
Hymenoptera	12,503	7,075	543.0	< 0.001*	348	270	976.0	0.0178*	3.655	3.817
Diptera	8,184	15,470	2,699.5	< 0.001*	219	203	2,332.5	< 0.001*	4.014	3.998
Coleoptera	2,772	4,054	2,175.5	0.0015*	348	306	957.5	0.0204*	4.429	3.422
Psocoptera	1,008	171	51.0	< 0.001*	19	13	46.5	< 0.001*	1.648	1.487
Blattaria	403	573	587.0	0.1223	12	9	299.0	0.0055*	1.427	1.033
Neuroptera	186	157	745.0	0.796	6	5	566.0	0.7742	0.861	0.9073
Orthoptera	72	150	1,112.0	0.001*	17	29	981.0	0.0065*	2.435	2.762
Thysanoptera	58	35	110.5	0.0992	1	1	–	–	0	0
Mantodea	13	10	90.0	0.5454	3	1	–	–	0.536	0
Odonata	3	5	–	–	1	4	–	–	0	1.332
Strepsiptera	2	3	–	–	1	1	–	–	0	0
Dermaptera	1	0	–	–	1	0	–	–	0	0
Phasmatodea	0	1	–	–	0	1	–	–	0	0
Total	48,338	46,275	1,429.0	0.462	1,087	932	1,422.5	0.5584	3.81	4.06

Coronel Pacheco, MG, 2010–2013. Comparison of abundance and richness was carried out using the non-parametric Wilcoxon matched-pairs test (exact *p* values).

*Orders with significant differences;

^–^Analysis not carried out due to low number of individuals or morphospecies.

When abundance, richness and diversity were assessed within each order, Hemiptera had a similar abundance, richness and Shannon’s H′ index in both systems (Table 1). Hymenoptera were significantly more abundant and species rich in the silvopastoral system, while Shannon’s H′ index was higher in the monoculture. Diptera were significantly more abundant in the monoculture and richness was significantly higher in the silvopastoral system, but Shannon’s H′ index was similar in both systems. Coleoptera were more diverse and significantly more species rich in the silvopasture, while abundance was higher in the monoculture. Psocoptera was significantly more abundant and species rich, with a higher value of Shannon’s H′ index in the silvopastoral system, while Orthoptera showed the opposite, being more abundant, rich and with a higher Shannon’s H′ index in the monoculture (Table 1). Blattaria was richer and had a higher Shannon’s H′ index in the silvopasture, but showed no difference in abundance. The other orders sampled, Neuroptera, Odonata, Mantodea, Strepsiptera and Thysanoptera had very low abundance and richness (Table 1) and did not differ significantly between systems. Dermaptera were present only in samples from the silvopasture, while Phasmatodea were only found in samples from the monoculture.

**Figure 1:**
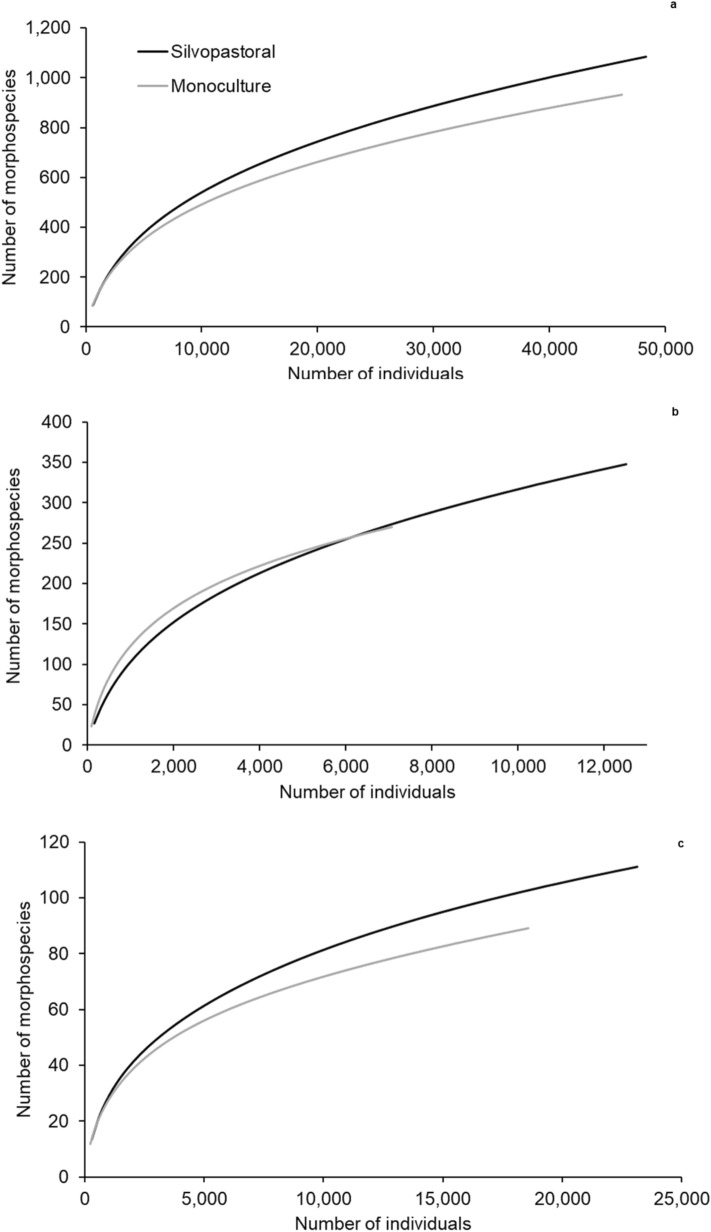
Rarefaction curves from the silvopasture and *Brachiaria decumbens* monoculture of (**a**) all morphospecies collected; (**b**) insects from the order Hymenoptera; and (**c**) insects from the order Hemiptera. Coronel Pacheco, MG, 2010–2013.

**Figure 2:**
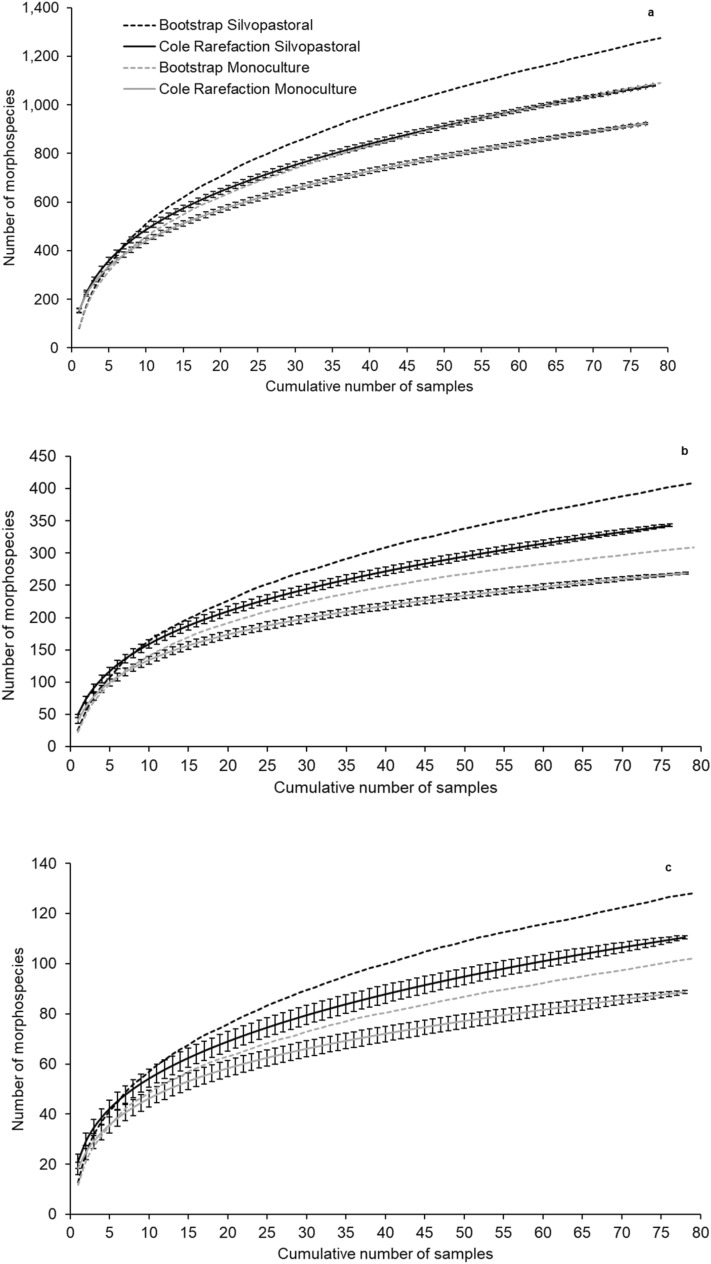
Coleman rarefaction curves and Bootstrap richness estimates from the silvopasture and *Brachiaria decumbens* monoculture for (**a**) all morphospecies collected; (**b**) insects from the order Hymenoptera; and (**c**) Insects from the order Hemiptera. Coronel Pacheco, MG, 2010–2013.

Analysis of the individual rarefaction curves for all the morphospecies revealed that the silvopasture accumulated more morphospecies than the *B. decumbens* monoculture (Fig. 1a). The Coleman rarefaction curves revealed that the silvopasture had a higher richness and species accumulation did not stabilise (Fig. 2a). The Bootstrap richness estimate (Fig. 2a) for the silvopastural system was 1,274 morphospecies and 1,089 for the monoculture, while the sampled richness was 1,087 and 932, respectively, indicating that we sampled approximately 85% of all morphospecies (Fig. 2a).

**Figure 3:**
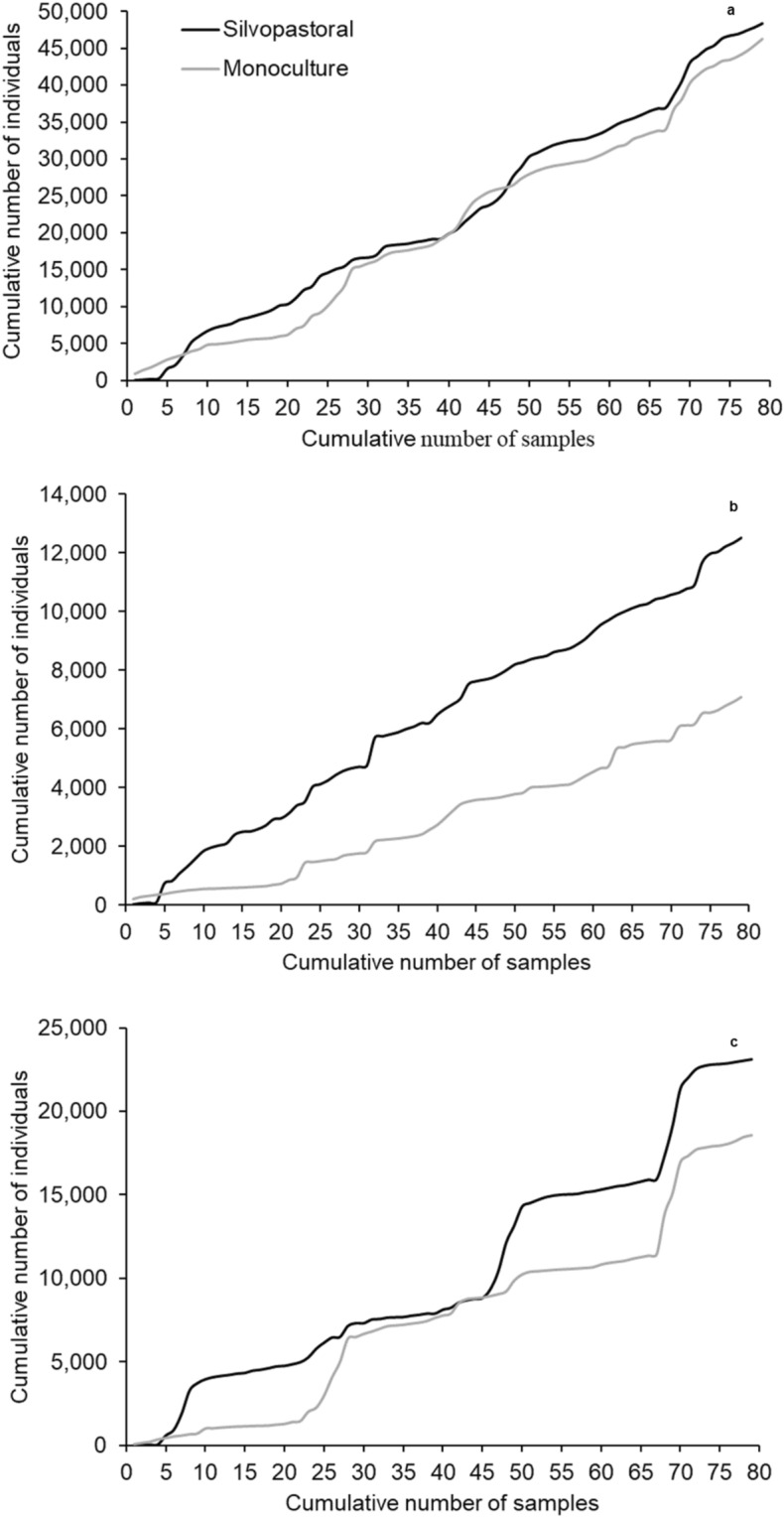
Individual accumulation curves from the silvopasture and *Brachiaria decumbens* monoculture of (**a**) all morphospecies collected; (**b**) insects from the order Hymenoptera; and (**c**) insects from the order Hemiptera. Coronel Pacheco, MG, 2010–2013.

The individual accumulation curves in both systems showed a faster accumulation in the silvopasture, with a small dip between sampes 41 and 47 (Fig. 3a), suggesting a higher abundance in the silvopasture.

**Figure 4:**
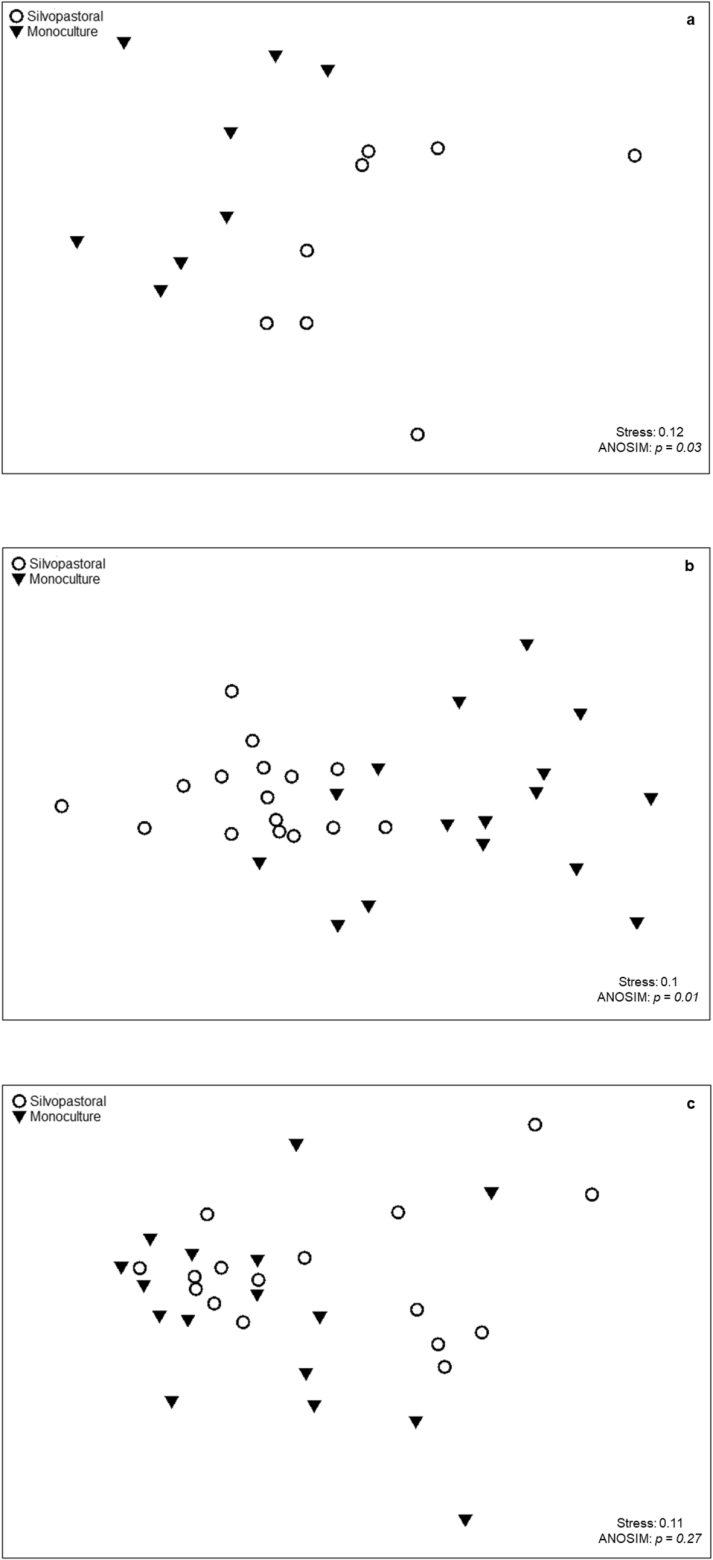
Graphical representation of the non-metric dimensional scaling (NMDS) with Bray–Curtis similarity and ANOSIM from the silvopasture and *Brachiaria decumbens* monoculture of (**a**) all taxa collected; (**b**) Insects from the order Hymenoptera; and (**c**) Insects from the order Hemiptera. Coronel Pacheco, MG, 2010–2013.

NMDS using the Bray–Curtis index showed a dissimilarity in composition between the monocultural and silvopastoral systems, with a clear separation between the two groups, with a 0.12 stress (Fig. 4a). Complementing the analysis, ANOSIM indicated a significant difference between the systems (p = 0.03). General dissimilarity between the systems was 63.71% according to SIMPER analysis.

### Hymenoptera fauna

To understand how families in the most abundant orders differ between the two systems we analysed the two most abundant orders, Hymenoptera and Hemiptera, which together contained 64% of the individuals collected. Of the order Hymenoptera, we collected 19,578 individuals, 12,503 from the silvopasture and 7,075 from the *B. decumbens* monoculture (Table 1, 2).

**Table 2:** Abundance, richness and Shannon’s H′ diversity of the insects in the order Hymenoptera collected in the silvopasture (S) and *Brachiaria decumbens* monoculture (M).

Family	Abundance				Richness				Shannon’s H′	
	S	M	V	*p* value	S	M	V	*p* value	S	M
Formicidae	11,313	4,740	390.5	< 0.001*	138	75	83.0	< 0.001*	3.212	2.672
Ichneumonidae	451	648	1626.5	0.0122*	65	41	1,216.5	0.3463	3.508	2.957
Braconidae	210	355	1,498.5	0.002*	20	18	1,068.0	0.1568	1.896	1.331
Pompilidae	97	236	1607.0	< 0.001*	26	30	1574.5	< 0.001*	2.659	2.531
Pteromalidae	75	127	527.0	0.2032	8	3	248.0	0.5360	1.346	0.6689
Vespidae	63	117	1,013.5	0.0072*	15	14	859.5	0.0040*	2.281	2.21
Chalcididae	59	155	900.5	0.001*	15	16	827.0	0.0040*	2.112	1.728
Ceraphronidae	45	177	1,271.0	< 0.001*	2	3	1,017	< 0.001*	0.5714	0.7115
Sphecidae	34	217	1812.5	< 0.001*	12	19	1729.0	< 0.001*	1.855	1.694
Eupelmidae	27	65	345.0	0.0192*	3	3	213.5	0.1428	1.071	1.071
Apidae	28	25	218.0	0.5496	9	9	207.0	0.5830	1.682	1.649
Evaniidae	26	15	61.0	0.0873	4	4	34.0	0.0152*	0.8817	0.7201
Mutillidae	19	96	688.0	< 0.001*	9	10	629.0	< 0.001*	1.908	1.602
Argidae	13	15	95.5	1,0000	4	5	90.0	0.8462	1.352	1.362
Halictidae	11	25	138.5	0.0624	2	3	130.0	0.1130	0.6555	1.021
Chrysididae	6	20	104.5	0.0550	3	4	108.0	0.0291*	0.8676	1.142
Scoliidae	6	20	155.0	0.0098*	4	7	160.0	0.0030*	1.242	1.751
Siricidae	5	3	–	–	2	1	–	–	0.5004	0
Dryinidae	4	5	–	–	1	2	–	–	0	0.673
Eucharitidae	4	9	–	–	1	3	–	–	0	0.6837
Stephanidae	3	0	–	–	2	0	–	–	0.6365	0
Gasteruptiidae	2	0	–	–	1	0	–	–	–	–
Torymidae	1	0	–	–	1	0	–	–	–	–
Diapriidae	1	0	–	–	1	0	–	–	–	–
Total	12,503	7,075	543.0	< 0.001*	348	270	976.0	0.0178*	3.655	3.817

Coronel Pacheco, MG, 2010–2013. Comparison of abundance and richness was carried out using the non-parametric Wilcoxon matched-pairs test (exact *p* values for Wilcoxon’s V statistic).

*Significant difference.

^–^Analysis not carried out due to low number of individuals or morphospecies.

In the silvopastoral system we found a total of 24 Hymenoptera families, while in the monoculture we found 20 families, all of which were found in the silvopasture (Table 2). The families found only in the silvopasture were the parasitoid families Stephanidae, Torymidae and Diapriidae as well as Gasteruptiidae, which are predators. Formicidae, Ichneumonidae, Braconidae and Pompilidae were the families with the highest abundance and richness in both systems. Formicidae was significantly more abundant in the silvopasture, while Pompilidae, Ichneumonidae and Braconidae were more abundant in the monoculture. Chalcididae, Ceraphronidae, Eupelmidae, Mutillidae, Sphecidae, Scoliidae and Vespidae were also significantly more abundant in the monoculture. Richness of Formicidae and Vespidae was significantly higher in the silvopasture, while richness of Pompilidae, Chalcididae, Ceraphronidae, Sphecidae, Mutillidae, Chrysididae and Scoliidae was significantly higher in the monoculture. Diversity was higher in the silvopasture than in the monoculture for Formicidae, Ichneumonidae, Braconidae, Pompilidae, Pteromalidae, Chalcididae, Sphecidae and Mutillidae, and was higher in the monoculture for Ceraphronidae, Halictidae, Chrysididae and Scoliidae (Table 2).

Individual rarefaction curves (Fig. 1b) show similar accumulation in both systems until 7,000 individuals, which was the abundance observed in the monoculture. The silvopasture continued accumulating morphospecies without stabilising. Coleman rarefaction for the monoculture (Fig. 2b) showed a greater stabilisation of species richness than in the silvopasture, suggesting sampling sufficiency. The Bootstrap estimate for the monoculture indicated that 87% of morphospecies were sampled (308 estimated morphospecies, compared to 270 observed morphospecies). In the silvopastoral system the Bootstrap estimate indicated that 85% of the morphospecies were sampled (408 estimated morphospecies, compared to 348 observed morphospecies), suggesting lower sampling sufficiency, but 29% higher observed richness.

The individual accumulation curve for Hymenoptera (Fig. 3b) showed higher individual accumulation in the silvopasture during the whole sampling period, with a steaper slope than the curve from the monoculture. Individual accumulation in the monoculture was slow at first (until sample 21), but then increased, with a few peaks, but always lower than the silvopasture.

Bray–Curtis NMDS analysis using abundance of Hymenoptera families showed partial overlap of the systems, suggesting some similarity in morphospecies composition, with a stress of 0.1 (Fig. 4b). ANOSIM, however, indicated that the systems are not similar (p = 0.01), while SIMPER indicated a general dissimilarity of 33.73% between the systems.

Analysis of Hymenoptera functional groups revealed that the most abundant were the omnivores, a group which was composed primarily of ants, with a relative abundance of 90% in the silvopastoral system and 68% in the monoculture (Table 3). Abundance and richness of this group were significantly higher, with a higher value of the Shannon’s H′ index, in the silvopasture. Abundance of parasitoids was lower in the silvopasture, with 1,034 individuals compared to 1,933 in the monoculture, and a relative abundance of 8% and 26%, respectively. Parasitoid richness was 165 morphospecies in the silvopasture, which was significantly greater than the 144 in the monoculture, and Shannon’s H′ was also higher in the silvopasture (Table 3). Pollinators and phytophages showed no significant difference in abundance or richness, while diversity was similar between the systems for pollinators and higher in the silvopasture for phytophages. Abundance and richness of predators was significantly higher in the monoculture (334 and 33, respectively) than in the silvopasture (99 and 28, respectively), while Shannon’s H′ index was higher in the silvopasture (2.823) than in the monoculture (2.522) (Table 3).

**Table 3:** Abundance, richness and Shannon’s H′ diversity for functional groups in the order Hymenoptera collected in the silvopasture (S) and *Brachiaria decumbens* monoculture (M).

	Functional group	Wilcoxon's matched pairs test				Relative Frequency (%)	
		S	M	V	*p* value	S	M
Abundance	Omnivores	11,313	4,740	390.5	< 0.001*	90.48	76.87
	Parasitoids	1,034	1,933	2,530.5	< 0.001*	8.27	16.77
	Pollinators	39	50	462.0	0.4762	0.31	0.81
	Phytophages	18	8	119.0	0.8117	0.14	0.13
	Predators	99	334	2041.0	< 0.001*	0.79	5.42
Richness	Omnivores	138	75	83.0	< 0.001*	39.66	27.78
	Parasitoids	165	144	2,211.0	0.0001*	47.41	53.33
	Pollinators	11	12	384.5	0.6043	3.16	4.44
	Phytophages	6	6	105.5	0.7207	1.72	2.22
	Predators	28	33	1,740.5	< 0.001*	8.05	12.22
Shannon’s H′	Omnivores	3.212	2.672				
	Parasitoids	4.301	3.924				
	Pollinators	1.987	2.028				
	Phytophages	1.706	1.586				
	Predators	2.823	2.522				

Coronel Pacheco, MG, 2010–2013. Comparison of abundance and richness was carried out using the non-parametric Wilcoxon matched-pairs test (exact *p* values for Wilcoxon’s V statistic).

*Significant difference.

### Hemiptera fauna

Over the 4-year sampling period, a total of 41,704 individuals were collected belonging to the order Hemiptera (23,133 from the silvopastoral system and 18,571 from the monoculture) (Table 1).We collected a total of 23 families, 22 from the silvopasture and 19 from the monoculture. Four families were exclusive to the silvopasture and one to the monoculture (Table 4). The families exclusive to the silvopasture were Aethalionidae, Alydidae, Delphacidae and Coreidae, while Acanoloniidae was the only family found exclusively in the monoculture. Cicadellidae was the most abundant family (91% of the individuals collected), but abundance did not differ significanty between the two systems. Cixiidae, Aphididae, Derbidae and Membracidae were significantly more abundant in the silvopasture, while Cercopidae was significantly more abundant in the monoculture. Richness of Cixiidae, Lygaeidae, Derbidae, Miridae and Membracidae was higher in the silvopasture, while Cercopidae richness was higher in the monoculture. The other families did not significantly differ between systems in abundance or richness (Table 4). Diversity was higher in the silvopasture for Miridae, while it was higher in the monoculture for Cicadellidae, Achilidae, Lygaeidae, Pentatomidae and Reduviidae (Table 4).

**Table 4:** Abundance, richness and Shannon’s H′ diversity for families in the order Hemiptera collected in the silvopasture (S) and *Brachiaria decumbens* monoculture (M).

Family	Abundance				Richness				Shannon’s H′	
	S	M	V	*p* value	S	M	V	*p* value	S	M
Cicadellidae	20,831	17,028	1,441.5	0.3932	31	24	1,436.0	0.6387	0.6246	0.7753
Cixiidae	1,196	552	494.0	< 0.001*	9	8	514.5	0.0010*	0.9519	1.059
Cercopidae	269	616	859.0	0.0004*	5	6	484.5	0.0360*	1.213	1.21
Achilidae	270	37	204.0	0.0610	5	5	151.5	0.0745	0.5635	1.344
Lygaeidae	149	82	709.0	0.0561	10	7	455.0	0.0540	0.8071	1.243
Aphididae	107	57	73.5	0.0093*	1	1	–	–	0	0
Derbidae	114	40	301.5	0.0020*	4	1	220.0	0.0001*	0.8859	0
Miridae	61	71	446.0	0.5610	12	10	223.5	0.0235*	1.883	1.351
Membracidae	50	24	184.5	0.0020*	10	8	186.0	0.0016*	1.271	1.272
Kinnaridae	28	8	10.0	0.1511	1	1	–	–	0	0
Anthocoridae	23	19	133.0	0.4170	2	1	–	–	0.1788	0
Tingidae	13	7	51.0	0.1884	4	4	51.0	0.1884	1.157	1.277
Pentatomidae	4	8	36.5	0.3583	2	4	–	–	0.5623	1.074
Delphacidae	4	0	–	–	4	0	–	–	1.386	0
Reduviidae	4	6	–	–	3	4	–	–	1.04	1.242
Fulgoridae	3	3	–	–	2	1	–	–	0.6365	0
Cydnidae	2	1	–	–	1	1	–	–	0	0
Aethalionidae	1	0	–	–	1	0	–	–	0	0
Cicadidae	1	1	–	–	1	1	–	–	0	0
Alydidae	1	0	–	–	1	0	–	–	0	0
Coreidae	1	0	–	–	1	0	–	–	0	0
Flatidae	1	10	–	–	1	1	–	–	0	0
Acanaloniidae	0	1	–	–	0	1	–	–	0	0
Total	23,133	18,571	1,286.5	0.1522	111	89	1,033.5	0.0565	1.143	1.219

Coronel Pacheco, MG, 2010–2013. Comparison of abundance and richness was carried out using the non-parametric Wilcoxon matched-pairs test (exact *p* values for Wilcoxon’s V statistic).

*Significant difference.

^–^Analysis not carried out due to low number of individuals or morphospecies.

Individual rarefaction curves for Hemiptera (Fig. 1c) showed faster morphospecies accumulation in the silvopastoral system than in the monoculture. The curves did not stabilise for either system. Coleman rarefaction (Fig. 2c) indicated that richness was significantly higher in the silvopastoral system than in the monoculture. For both systems the curves did not stabilise, and the Bootstrap estimates were 127 morphotypes in the silvopasture and 102 in the monoculture, compared to 111 and 89 observed morphotypes, respectively (approximately 87% of the estimated species in both systems).

Individual accumulation curves in the silvopasture began with peaks of collection during the first eight samples, followed by a continuous accumulation and a new peak between samples 44 and 48, and then continuous growth until a new peak between samples 66 and 72, and then constant accumulation until the end of sampling. Despite the variation, accumulation in the silvopasture was higher than in the monoculture. In the monoculture, accumulation was stable until sample 21, and then increasing until sample 27, after which we observed a continuous accumulation until sample 67, after which accumulation was similar to the silvopasture until the end of sampling (Fig. 3c).

NMDS of the abundance of Hemiptera using Bray–Curtis suggested similarity in the composition of Hemiptera families in the two systems, since there was no separation between the samples and a complete overlap of the symbols used for monuculture and silvopasture, even with low stress (0.11 Fig. 4c). Using ANOSIM no significant difference was detected (p = 0.27). Dissimilarity as measured by SIMPER was 28.67%.

When the Hemiptera families were separated into functional groups, phytophages, omnivores and predators were found, but the only difference detected between the systems was the richness of omnivores, which was higher in the silvopasture with 12 morphotypes compared to 10 in the monoculture. The abundance of phytophages represented 99.5% of the individuals collected. No other significant differences were detected in abundance or richness, and Shannon’s H′ index was higher in the silvopasture for omnivores and higher in the monoculture for predators (Table 5).

**Table 5:** Abundance, richness and Shannon’s H′ diversity functional groups in the order Hemiptera collected in the silvopasture (S) and *Brachiaria decumbens* monoculture (M).

	Functional group	Wilcoxon's matched pairs test				Relative frequency (%)	
		S	M	V	*p* value	S	M
Abundance	Phytophages	23,042	18,473	1,261.5	0.1654	99.61	99.47
	Omnivores	61	71	486.5	0.5473	0.26	0.38
	Predators	30	27	9,243.5	0.4974	0.15	0.15
Richness	Phytophages	93	73	1,091.0	0.0530	83.78	81.11
	Omnivores	12	10	254.0	0.0255*	10.81	11.11
	Predators	6	7	204	0.3478	5.41	7.78
Shannon’s H′	Phytophages	1.113	1.183				
	Omnivores	1.883	1.351				
	Predators	0.9784	1.102				

Coronel Pacheco, MG, 2010–2013. Comparison of abundance and richness was carried out using the non-parametric Wilcoxon matched-pairs test (exact *p* values for Wilcoxon’s V statistic).

*Significant difference.

## Discussion

The adoption of silvopastures has the potential to minimise the environmental impacts of livestock^13,27^. To make this possible, we need to understand how different livestock systems affect biodiversity and ecological processes that help the system to function. The study of “superior” taxonomic categories (Orders) allows understanding of large changes in the biodiversity associated with different productive systems. In this study we demonstrated changes in the abundance and species richness of several insect orders, as well as changes in community composition, even though no differences were observed in overall abundance, richness or diversity. These compositional changes could result from changes in abiotic conditions in the two environments, or from a shift from more generalist species in the simpler environment towards more specialists in the more complex environment, without a change in richness or abundance^28,29^. This substitution of generalists with specialists is well known in environmental restoration^30^ and there is evidence that simplified pastures contain a greater proportion of specialist species^31,32^. Since insects have diverse functional roles in agroecosystems as phytophages, predators, detritivores, and pollinators^33^, these changes in composition can have an important influence on ecological functions in the pasture ecosystem.

Within the major insect orders the silvopasture showed higher abundance of Hymenoptera and Psocoptera. Richness was higher in the silvopasture for Hymenoptera, Diptera, Coleoptera, Psocoptera and Blattaria while diversity was higher in the silvopasture for Coleoptera, Psocoptera and Blattaria. The increase in richness and diversity in the silvopasture could be due to the presence of more specialist species that are sensitive to changes in land use^34^. More elaborate management^35^ allows for greater complexity of vegetation structure, leaf litter and soil conditions that create greater microhabitat diversity that can be colonised by a greater number of species^36^. It is also known that tree vegetation and the presence of native vegetation^37,38^ increase the richness of several groups that depend on above-ground vegetation and feed as predators or on pollen^39^ or as parasitoids^40^. In silvopastoral systems with *Acacia*, richness of certain groups was similar to neighbouring native forests^9^ and these systems contain greater richness than conventional pastures^41^. Not all groups responded with increases in the silvopasture. In the monoculture, Diptera, Coleoptera and Orthoptera abundance were higher as was Orthoptera richness and diversity. Higher abundance of Diptera in pasture monocultures has been reported previously^42,43^. Composition of Orthoptera communities is known to be greatly affected by plant cover^44^ and changes of environmental conditions, forming distinct communities characteristic of areas with low vegetational cover and others characteristic of high vegetation cover^45–47^. Some studies have reported that abundance of some groups falls with habitat simplification^48^, while in other groups abundance increases^49^ which was also recorded in our study. The sensitivity of many of these species to environmental conditions has led to several groups being recognised as bioindicators of conditions in agricultural systems, native ecosystem degradation and ecological restoration^50–53^.These studies, along with others in traditional silvopastoral systems^54^ highlight the importance of promoting these systems for livestock farming allied with biodiversity conservation and conservation of ecosystem services, though care must be taken, since the effects can depend on the design of the system and tree species chosen^55^.

The importance of species in the order Hymenoptera that are beneficial for the functioning of the productive system through either pollination or pest regulation is well documented^56,57^. The observed and estimated richness in our systems agree closely with other studies where between 70–80% of the estimated richness was sampled^58,59^. The difference in Bootstrap estimates of 100 more species in the silvopasture shows a large impact of the conversion from monoculture to silvopasture on Hymenoptera, increasing its species richness by 29%. The NMDS results for Hymenoptera showed that in the monoculture it had a more heterogeneous and disperse composition compared to the silvopasture. This is probably due to its greater sensitivity to climatic and seasonal variations in the monoculture and, therefore, lower resilience^60^, causing greater temporal oscillations, which could potentially threaten system stability^60–62^. The increase in richness within these groups of beneficial organisms can have an important effect beyond the silvopastoral system, since it has the potential to improve biological control and crop pollination at a larger scale^56,63^. Due to this importance of Hymenoptera, conversion of monocultures to silvopastures, if implemented at larger scales, has the potential to positively influence the number of beneficial insects at landscape scales and benefit not only the silvopasture itself, but also other production systems in the region through spillover effects. There is evidence that different groups of Hymenoptera respond differently to land-use changes^64,65^ and some groups are useful indicators of environmental degradation^15,19^.

Formicidae was the most abundant Hymenoptera family in both systems. For this family abundance, richness and diversity were significantly higher in the silvopasture, as has been found previously^15,66–68^. In the silvopastoral system this increase in ant diversity could be due to the differences in tree physiology and structural complexity, resulting in an increase in diversity^69,70^. Many ant species nest in tree canopies, trunks or branches, as well as dry branches that fall to the ground, and tree presence increases ant activity in agroforestry systems^71^. Due to their abundance and diversity ants play an important role as predators of herbivores^72^. Simplified pastures tend to have a greater number of generalist and opportunist species, while more complex environments have a greater proportion of specialist ants that carry out a greater range of ecological functions^32^. The most abundant functional group were the omnivores, which contain many Formicidae. This group also showed higher abundance, richness and H′ in the silvopasture, where diversification offers complementary habitats and a greater range of food resources^73^, stimulating greater diversity and abundance of many taxa^73–75^. In contrast, Vespidae were more abundant in the monoculture. This family is mainly composed of important predators of Lepidoptera larvae^76,77^. The abundance and richness of Pteromalidae, Sphecidae and Mutillidae were higher in the monoculture, but diversity was higher in the silvopasture. The same was observed when Hymenoptera were separated into the functional groups and predators, which contains Mutillidae and Sphecidae, were also more abundant and species rich in the monoculture, while diversity was higher in the silvopasture. Parasitoid families, such as Ichneumonidae, Braconidae, Pteromalidae and Eupelmidae were more abundant in the monoculture, while Chalcididae was more abundant and species rich in the monoculture. Apart from Eupelmidae, all of these families were, however, more diverse in the silvopasture. These results differ slightly to those observed by Auad, et al.^15^, who analysed Hymenoptera in the same system for a shorter time period and found greater abundance of Icheumonidae and Braconidae in the silvopasture as well as to other studies where abundance of parasitoids was higher in pastures with lower management intensity, and lower in conventional monocultural pastures^28^. Our overall analysis of parasitoids as a functional group showed greater richness and diversity in the silvopasture, but greater abundance in the monoculture, very similar to that reported previously^15^. Auad, et al.^15^ also found greater Shannon’s H′ in the silvopasture for several parasitoid families, as found here with a longer sampling period for sevral parasitoid families and for parasitoids as a group.

Most Hemiptera species are phytophagous, and feed from the phloem, xylem or by piercing plant cells. Therefore, the silvopasture provides the greater availability of plant tissues and feeding sites that result from the greater structural complexity of the silvopastoral system. It is known that the community of Hemiptera is affected by the vegetational complexity of pastures^78^, as well as by the implementation of agroforesty systems, where the abundance and richness of some Hemiptera groups are related to the presence of certain tree species that compose the system^79^. However, NMDS and ANOSIM suggested high similarity between the systems, with a high overlap of the symbols used for monoculture and silvopasture and a lack of discrete groups. The peaks of accumulation observed during the four years of sampling correspond with the peaks in leafhoppers of the family Cicadellidae. The high observed abundance of Cicadellidae in pastures is well documented^80,81^, and this family can account for over 30% of arthropod abundance in pastures, and over 50% of Hemiptera^78,82,83^. Cercopidae was also among the most abundant Hemiptera families, but was more abundant and rich in the monoculture. This is the family responsible for the most significant damage to pastures, since several species are important pests in livestock production systems throughout tropical America^84,85^. Achilidae, Cixiidae, Derbidae and Membracidae consist mainly of phloem-feeding species, feeding on both underground and aboveground tissues^86,87^. Many species in these families form mutualistic associations with ants, where they provide honeydew in exchange for protection against predators and parasitoids^88^. In the silvopasture there was an increase in abundance of these families, which corresponded with an increase in ant abundance and richness. It is therefore likely that the increase in these species is associated with an increase in ant abundance and the frequency of interaction with ants. These mutualistic interactions are likely to be common in the silvopasture, and the increase in this interaction can, in turn, affect the efficiency of ants as predators in the system, since it is known that the presence of this interaction increases predation and reduces oviposition by other insects^89–93^. The hypothesis that the complexity of ecological interactions in silvopastures is greater deserves further attention.

Hence, in general we observed important differences in the insect fauna at the order level, and the conversion of *B. decumbens* monocultures to a silvopastoral system affected the abundance, richness and diversity of the orders Hymenoptera, Diptera, Coleoptera, Psocoptera, Blattaria and Orthoptera, despite not having affected overall abundance richness and diversity. Furthermore, our results show that system conversion from monoculture to silvopasture affects the abundance and diversity of insects in the order Hymenoptera. These changes can be measured through changes in one or more of the indices in the families Formicidae, Ichneumonidae, Braconidae, Pompilidae, Vespidae, Chalcididae, Ceraphronidae, Sphecidae, Eupelmidae, Evaniidae, Mutillidae, Chrysididae and Scoliidae. These families can therefore be considered candidates for bioindicators of changes to the system. Formicidae, which had higher abundance, richness and diversity in the silvopasture, and the family Pompilidae, which had higher abundance and richness in the monoculture but higher diversity in the silvopasture, are excellent candidates for bioindicators. The changes in the system had a smaller effect on the composition of species in the order Hemiptera. Differences were observed for overall phytophage richness, species richness of Cixiidae, Cercopidae, Derbidae, Miridae and Membracidae, and in the abundance of the families Cixiidae, Cercopidae, Aphididae, Derbidae and Membracidae. None of the Hemiptera demonstrated potential as bioindicators of conversion of pastures to silvopastures.

## Conclusions

We provide evidence that the insect fauna changes upon conversion of the *B. decumbens* monoculture to a silvopastoral system. Since sustainability of pastures depends upon of organisms that play important roles in maintaining ecological systems, among these the insects, measures should be taken to encourage the implementation of silvopastures on a larger scale.

## Materials and methods

### Study system

This study was carried out at the *Centro de Pesquisa da Embrapa Gado de Leite*, in the city of Coronel Pacheco, Minas Gerais State, Brazil (21° 33′ S, 43° 6′ W). The city has a rainy tropical climate, with an average annual rainfall of 1533 mm, 19.5 °C average annual temperature with an amplitude of over 5 °C, rainy summers and dry winters, between June and September.

The experiment was carried out in two 4 ha areas. One was a *Brachiaria decumbens* monocultural pasture. The second area was a silvopastoral system composed of 30 m wide strips of *B. decumbens* alternated with 10 m wide strips of the trees *Acacia mangium* Willd and *Eucalyptus grandis* W. Hill ex Maiden planted in a 3 m × 3 m spacing.

### Sampling and identification

Insect sampling was carried out during four years, from January 2010 to December 2013, using Townes model Malaise traps^94^, and one trap in each area. The trap in the silvopastoral system was located in one of the tree strips in the middle of the experimental area, geographical coordinates 21° 33′ 10′ S and 43° 15′ 15′ W. The trap in the monoculture system was located at 21° 33′ 07″ S and 43° 15′ 17″ W. The two traps were run continuously and concurrently during the entire 4-year period to be completely comparable. These traps were inspected every 15 days, and the captured insects were preserved in 70% alcohol. Each 15-day period from each site therefore constituted a single sample. The samples were taken to the Entomology Laboratory of *Embrapa Gado de Leite* in the city of Juiz de Fora, Minas Gerais, Brazil, where they were stored in jars with 70% alcohol for identification. The insects in the samples were separated into different orders and families and identified as morphospecies with the aid of entomological keys^95–97^ (except for insects in the order Lepidoptera, as well as micro-Hymenoptera).

The morphospecies in the most abundant orders, Hymenoptera and Hemiptera, were separated into the following functional groups using the morphological characters of each morphospecies as well as the dominant characters of each family: predators, parasitoids, omnivores, phytophages and pollinators. The samples are catalogued in the inventory of the Entomology Laboratory of *Embrapa Gado de Leite*.

### Statistical and faunistic analyses

We carried out these faunistic analyses at different levels of taxonomic refinement. First, we analysed abundance, richness and diversity of the overall insect fauna. To do this, we compared the total abundance (based on the number of specimens sampled) between the sites and calculated the total richness and Shannon diversity for each site (based on the number of morphospecies). Second, for each order we compared the total abundance within the order between the sites and calculated the total richness and Shannon diversity for each order in each site. Third, for Hymenoptera and Hemiptera we compared the total abundance within each family of these orders between the sites and calculated the total richness and Shannon diversity for each family within each site. For each functional group within Hymenoptera and Hemiptera (see above) we compared the functional group abundance between the two sites and calculated the functional group richness and Shannon diversity for each functional group within each site.

For each sample we measured the abundance of each morphospecies present. This allowed us to calculate a per-sample abundance and richness that were compared between the monoculture and silvopasture using Wilcoxon’s matched pairs (signed-rank) test using R^98^, at significance of 0.05. We used a paired analysis, where our samples were paired by date. Due to trampling by cattle and bad weather we lost a total of 29 samples. When a sample was lost, the corresponding pair was removed from the analysis in order not to unbalance the analysis, leaving a total of 158 samples (79 sample pairs) that were analysed. Total species richness was calculated from the number of morphospecies collected, pooling all samples, by generating individual accumulation curves and Coleman rarefaction curves^99^, as well as estimating Bootstrap richness estimators in EstimateS^®^^100^. Overall diversity was calculated by generating Shannon–Wiener’s diversity index (H′)^101^ from the total abundance of each morphospecies in the monoculture and silvopasture. In order to analyse community composition, we applied nonmetric multidimensional scaling (NMDS)^102^, using the Bray–Curtis similarity index; analysis of similarities (ANOSIM), following Clarke^103^; and SIMPER in Primer v7^104^. To do this, first we standardised abundances to calculate relative abundance of each morphospecies. Second, we square-root transformed each value. Using these we generated a Bray–Curtis similarity matrix and carried out the NMDS.

## Data Availability

The datasets generated and/or evaluated during the current study are available from the corresponding author on request.
